# Characterisation of the Mouse Vasoactive Intestinal Peptide Receptor Type 2 Gene, *Vipr2*, and Identification of a Polymorphic LINE-1-like Sequence That Confers Altered Promoter Activity

**DOI:** 10.1111/j.1365-2826.2006.01498.x

**Published:** 2007-01

**Authors:** G Steel, E M Lutz

**Affiliations:** Strathclyde Institute of Pharmacy and Biomedical Sciences, University of Strathclyde Royal College, Glasgow, UK

**Keywords:** vasoactive intestinal peptide (VIP), pituitary adenylate cyclase activating polypeptide (PACAP), G protein-coupled receptor, gene, LINE-1

## Abstract

The VPAC_2_ receptor is a seven transmembrane spanning G protein-coupled receptor for two neuropeptides, vasoactive intestinal peptide (VIP) and pituitary adenylate cyclase-activating polypeptide (PACAP). It has a distinct tissue-specific, developmental and inducible expression that underlies an important neuroendocrine role. Here, we report the characterisation of the gene that encodes the mouse VPAC_2_ receptor (*Vipr2*), localisation of the transcriptional start site and functional analysis of the promoter region. The *Vipr2* gene contains 12 introns within its protein-coding region and spans 68.6 kb. Comparison of the 5′ untranslated region sequences for cloned 5′-RACE products amplified from different tissues showed they all were contained within the same exon, with the longest extending 111 bp upstream of the ATG start site. Functional analysis of the 3.2-kb 5′-flanking region using sequentially deleted sequences cloned into a luciferase gene reporter vector revealed that this region is active as a promoter in mouse AtT20 D16:16 and rat GH4C1 cell lines. The core promoter is located within a 180-bp GC-rich region proximal to the ATG start codon and contains potential binding sites for Sp1 and AP2, but no TATA-box. Further upstream, in two out of three mice strains examined, we have discovered a 496-bp polymorphic DNA sequence that bears a significant identity to mouse LINE-1 DNA. Comparison of the promoter activity between luciferase reporter gene constructs derived from the BALB/c (which contains this sequence) and C57BL/6J (which lacks this sequence) *Vipr2* promoter regions has shown three-fold difference in luciferase gene activity when expressed in mouse AtT20 D16:16 and αT3-1 cells, but not when expressed in the rat GH4C1 cells or in COS 7 cells. Our results suggest that the mouse *Vipr2* gene may be differentially active in different mouse strains, depending on the presence of this LINE-1-like sequence in the promoter region.

The 28 amino acid vasoactive intestinal peptide (VIP) and 38 amino acid pituitary adenylate cyclase-activating polypeptide (PACAP) are structurally related neuropeptides that have several overlapping and distinct functions in the central nervous system (CNS) and periphery (1–5). Three genes have been cloned that encode distinct VIP/PACAP receptor proteins belonging to the class II secretin receptor G protein-coupled receptor (GPCR) family (6–8). Two are receptors at which VIP and PACAP are equipotent and have been designated the VPAC_1_ and the VPAC_2_ receptors. The third receptor, at which PACAP ≥ 100-fold more potent than VIP, has been designated the PAC_1_ receptor (9). Further diversity in VPAC/PAC_1_ receptor isoforms is generated through alternative splicing of their respective genes, including several N-terminal and intracellular loop 3 splice variants of the PAC_1_ receptor (10–14), and the recently identified Δ exon 12 variant of the VPAC_2_ receptor (15). Similar to other members of the class II secretin receptor GPCR family, the VPAC/PAC_1_ receptors couple to adenylate cyclase activation and are capable also of coupling to additional second messenger pathways through distinct interactions with different heterotrimeric and small G proteins (16). The diversity in receptor isoforms generated by alternative splicing is reflected by altered ligand activation and G protein coupling for the VPAC/PAC_1_ receptors. For example, there are notable differences in the activation of certain N-terminal domain PAC_1_ receptor splice variants by VIP (14) and for the PAC_1-hop-1_ but not the PAC_1-null_ intracellular loop 3 isoforms; coupling to phospholipase D activation involves an additional ADP ribosylation factor-dependent pathway (17, 18). In addition, the VPAC_2_ receptor Δ exon 12 variant does not couple to adenylate cyclase activation (15). These differences may underlie the various actions of VIP and PACAP in different tissues and cell-types.

The distinct expression profile of the VPAC_2_ receptor in the CNS and also in several endocrine cell types and tissues (7, 19–23) has prompted the suggestion that the VPAC_2_ receptor may be responsible for mediating the neuroendocrine effects of VIP (19). In the pituitary, VPAC_2_ receptor expression levels are up-regulated during pregnancy (19), where one of its roles may be to mediate the potent prolactin-releasing action of VIP. The VPAC_2_ receptor also is expressed in several pituitary-derived clonal cell lines including rat somatomammotroph GH3 and GH4C1 cells, mouse adrenocorticotroph AtT20 and gonadotroph αT3-1 cells (21, 24, 25) suggesting that, in addition to its role in mediating VIP and PACAP regulation of the synthesis and release of different pituitary hormones, it may have a role also in the development of pituitary cell lineages. In support of this, expression is detected quite early in development, and transcripts encoding VPAC_2_ receptors are expressed in mouse embryonic stem cells (26, 27) and mouse embryonic brain tissue as early as E9.5 (28). In the adult brain, mRNA encoding the VPAC_2_ receptor is present at relatively high levels in the suprachiasmatic nucleus, thalamus, hippocampus, olfactory bulb, and several of the hypothalamic nuclei, in agreement with the mapping of VPAC_2_ receptor binding sites using a receptor-specific ligand (29). The VPAC_1_ receptor is constitutively expressed in a number of different immune cell types whereas expression of the VPAC_2_ receptor is induced in certain monocytes and lymphocytes only after activation (30–32), suggesting that the VPAC_1_ and VPAC_2_ receptors have different roles in mediating the anti-inflammatory and immunomodulatory actions of VIP and PACAP (32). Expression of the VPAC_2_ receptor is induced also in spinal glial cells following a chronic constrictive nerve injury (33) where it participates in sensory sensitisation involving p38 MAP kinase activation (34).

Very little is known about the regulatory elements involved in the tissue-specific, inducible, and developmentally regulated expression of the gene encoding the VPAC_2_ receptor. Previously, we reported the characterisation of the human VPAC_2_ receptor gene and identification of a putative proximal promoter region (35). In the present study, we have characterised the mouse VPAC_2_ receptor gene, *Vipr2*. The transcriptional start sites were determined by 5′-RACE polymerase chain reaction (PCR) analysis utilising several anchored cDNA libraries derived from different mouse tissues and embryonic stages. In addition, the promoter activity and core promoter region within 3.2 kb of 5′-flanking sequence were characterised using luciferase reporter gene constructs transiently expressed in different pituitary cell lines. Recent research has suggested that polymorphisms of the human *VIPR2* gene promoter may play a role in autism by altering transcriptional regulation and the level of protein expression (36). Here, we have identified a polymorphic LINE-1 (L1)-like sequence that is present in the *Vipr2* promoter region in 129 and Balb/c but not in C57Bl/6J and show that this sequence confers increased expression levels of a luciferase reporter gene.

## Materials and methods

### Materials

Tissue culture media and Lipofectamine 2000 were obtained from Invitrogen (Paisley, UK); Primocin from Autogen Bioclear UK Ltd (Calne, UK); Genejuice from Novagen, Merck Biosciences Ltd (Nottingham, UK); standard laboratory chemicals of Analar grade were obtained from Sigma or BDH Chemicals Ltd (Poole, UK); oligonucleotide primers were obtained from Oswel DNA Service (Southampton, UK) and Life Technologies (Paisley, UK).

### Anchored 5′-RACE (rapid amplification of cDNA ends) for the mouse VPAC_2_ receptor cDNA

Total RNA was isolated from the mouse adrenocorticotroph AtT20 D16:16 cell line using Catriomox-14 surfactant reagent (VH BIO Ltd, Newcastle-upon-Tyne, UK). Poly A+ RNA was isolated from AtT20 total RNA with the Qiagen Oligotex kit and an anchored cDNA library synthesised from 1 µg of poly A+ RNA using the Clontech Marathon cDNA Amplification kit (BD Biosciences, Oxford, UK). The 5′ end of the mouse VPAC_2_ receptor was amplified using the Clontech anchor primer AP1, and the VPAC_2_ receptor specific primer exon4.rp (5′-ATGTCTCTGACCATCCATCGC-3′), in a 35-cycle touchdown PCR reaction with Advantage KlenTaq Polymerase Mix (BD Biosciences) according to the manufacturer's guidelines. Amplification products were checked by gel electrophoresis and Southern blotting. The first round amplification reaction then was diluted 1 : 100 µl with sterile H_2_O, and a second round of PCR was performed under the same conditions using 5 µl of diluted first round mix together with the Clontech nested primer AP2 and the mouse VPAC_2_ receptor specific primer exon1.rp (5′-CAGCAACCAGCAGTAGCAGGTCAGCACCAC-3′). Total RNA was isolated from olfactory bulb tissue from BALB/c and from C57BL/6J mouse strains with the Wizard RNA kit (Promega, Southampton, UK). Anchored cDNA libraries were synthesised from 1 µg total RNA using the Clontech SMART RACE cDNA amplification kit (BD Biosciences) and Superscript II (Invitrogen). The 5′ end of the mouse VPAC_2_ receptor was amplified with the Clontech anchor primer 5′UPM and the VPAC_2_ receptor specific primer exon11.rp (5′-GCCAAACAGGGGGATTAGCAGCAG-3′) using the HF2 Advantage PCR kit (BD Biosciences) and the touchdown PCR method. The final amplification products were size selected by gel electrophoresis, subcloned into the pGEM-T Easy vector (Promega) and sequenced in both directions.

Sure-RACE mouse panels (Origene Technologies Inc., Cambridge Bioscience, Cambridge, UK) were used as described by the manufacturer but with the Advantage-GC2 kit (BD Biosciences) and Taq DNA polymerase (Stratagene Europe, Amsterdam, the Netherlands). For first round amplification, the Sure-RACE anchor primer ADP1 and the VPAC_2_ receptor specific primer exon5.rp (5′-GCCCAAGGTATAAATGGCCTTC-3′) were used in a 20-cycle touchdown PCR reaction. The first round amplification reaction then was diluted 1 : 100 µl with sterile H_2_O, and a second round of PCR was performed under the same conditions using 5 µl of diluted first round mix together with the Sure-RACE nested primer ADP2 and the mouse VPAC_2_ receptor specific primer exon3.rp (5′-CTGAATACTTTGGGGCAGGG-3′). The second round amplification products were separated by agarose gel electrophoresis and visualised following staining with ethidium bromide with a uv light box. Amplification products were isolated following gel electrophoresis, subcloned into the pCR4-TOPO vector using the TOPO TA cloning kit (Invitrogen) and recombinant plasmids were sequenced.

### Reporter plasmids used in expression studies

A genomic DNA clone (λESD1) containing the *Vipr2* exon 1, intron 1 and exon 2 along with the 5′ flanking sequence was isolated previously from a λ2001 genomic DNA library made from mouse strain 129-derived ES cell DNA (provided by Dr A. J. H. Smith, Centre for Genome Research, University of Edinburgh, Edinburgh, UK) and the 5.6-kb *Xba*I fragment spanning this region subcloned into pGEM11z (Promega). The 3.2-kb *Xba*I/*Sfo*I fragment spanning the 5′ sequence and part of exon 1 prior to the translational start site (−3300/−33) was subcloned into the reporter plasmid pGL3-Basic (Promega). Deletion mutants were created by subcloning smaller fragments generated using restriction enzyme sites, including the 2.5-kb *Nhe*I/*Sfo*I (−2514/−33), 1.6-kb *Hind*III/*Sfo*I (−1642/−33), 0.55-kb *Nco*I/*Sfo*I (−584/−33), 0.18-kb *Bgl*II/*Sfo*I (−212/−33) and 3-kb *Xba*I/*Bgl*II (−3300/−213) constructs. The 2.2-kb *Sfo*I*/Xho*I fragment (−32/+2167) was also subcloned into pGL3-Basic. The C57BL/6J and BALB/c reporter constructs were created by amplifying genomic DNA isolated from each mouse strain with the oligonucleotide primer pair 15075 (5′-GTAGCCCCTAATGGGTCGAGTGT-3′) and 8484 (5′-GCACCAGCAACCAGCAGTAG-3′) using the GC-melt genomic PCR kit (BD Biosciences). Amplification products were gel purified, subcloned into pGEMTeasy (Promega) and verified by sequencing. The *Spe*I/*Sfo*I fragments from each were then subcloned into pGL3-Basic.

### Cell culture and transfection

Mouse adrenocorticotroph AtT20 D16:16 cells were maintained in Dulbecco's modified Eagle medium (DMEM) containing 10% fetal calf serum (FCS) and 5 µg/ml Primocin. Mouse gonadotroph αT3-1 cells were maintained in DMEM supplemented with 100 µm pyruvate, 5 µg/ml Primocin and 10% FCS. Rat somatomammotroph GH4C1 cells were maintained in Ham's F10 medium supplemented with 15% horse serum, 2.5% FCS and 5 µg/ml Primocin. COS 7 cells were maintained in DMEM containing 10% normal calf serum and 5 µg/ml Primocin. One day prior to transfection, cells were trypsinised and 1 × 10^5^ cells/well plated into 24-well plates. αT3-1 cells were transfected using Lipofectamine 2000 and AtT20, GH4C1 and COS 7 cells were transfected using GeneJuice according to the manufacturer's guidelines. Briefly, the transfection reagent to plasmid DNA was used in a ratio of 3 : 1 and diluted in OptiMEM. Cells were cotransfected with a 3 : 1 mixture of the experimental pGL3 reporter construct and the control pRL-TK plasmid encoding *Renilla* luciferase (Promega) for normalisation.

### Reporter gene assays

Luciferase activity was measured with the Dual-Glo Luciferase Assay System (Promega). Culture medium was removed from transfected cells in each well and replaced with 75 µl of DMEM along with 75 µl of Dual-Glo Luciferase Reagent. Samples were incubated for 10 min at room temperature, then transferred to a white 96-well plate (Costar, Fisher Scientific, Loughborough, UK). Firefly luciferase activity was measured with a BMG Labtech LUMIstar Galaxy Plate Reader (BMG Labtech Ltd, Aylesbury, Bucks, UK). Immediately afterwards, 75 µl of *Renilla* luciferase reagent diluted in Stop-Glo buffer was added to each well, and the plate incubated for 10 min at room temperature before measuring *Renilla* luciferase activity.

## Results

### The mouse VPAC_2_ receptor is encoded by 13 exons

**Fig. 1:**
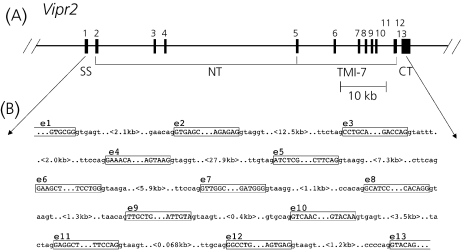
(a) Schematic diagram of the gene encoding the mouse VPAC_2_ receptor, *Vipr2*. The exons encoding the signal peptide sequence (SS), amino-terminal domain (NT), seven transmembrane domains (TM1-7) and the carboxyl tail (CT) are indicated underneath. (b) The sequence of the exon-intron boundaries and the size of each intron.

The organisation of the mouse *Vipr2* gene was determined by alignment of the mouse VPAC_2_ receptor cDNA sequence (37) with the Genebank deposited genomic DNA sequences of the mouse chromosome 12 clone RP24-386J17 (accession number AC105950), the C57BL/6J clone RP23-32J01 (accession number AC073564) and the DNA sequence obtained for genomic clones encoding the mouse *Vipr2* previously isolated through the screening of a λ2001 mouse 129 strain embryonic stem cell-derived genomic library (Mackay, West, Lutz and Harmar, unpublished data; the ES-129 *Vipr2* gene sequence has been deposited in the EMBL database under the accession numbers AJ272314-AJ272323) The protein coding region of the mouse *Vipr2* gene (Fig. 1a) is comprised of 13 exons, with the ATG translational start site located in exon 1 and the stop codon and polyadenylation signal in exon 13. The gene spans approximately 68.6 kb and is interrupted by 12 introns, ranging from 68 bp (intron 11) to 28 kb (intron 4) in size (Fig. 1b). The N-terminal extracellular domain is encoded by four exons, the transmembrane spanning domains by eight exons, and the C-terminal tail by one exon.

### Multiple transcriptional start sites are located within the first coding exon of Vipr2

**Fig. 2:**
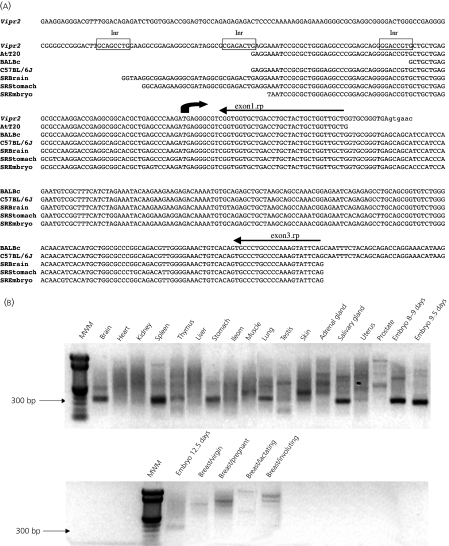
(a)Alignment of sequences derived from different cloned 5′ RACE products with the *Vipr2* exon 1 and 5′ flanking genomic sequence. The sequences shown represent the longest products amplified from each anchored cDNA library, derived from AtT20 D16:16 cells, BALB/c and C57BL/6J olfactory bulb tissues or the RACE panel shown in (b). The positions of putative Inr elements are indicated by the boxed sequence and the translational start site is indicated by the dark arrow. The lower case letters indicate intron 1 sequence. (b) 5′ RACE analysis of a multitissue RACE panel derived from 24 mouse tissues and developmental stages. Nested polymerase chain reaction was performed to amplify the 5′ ends of the mouse VPAC_2_ receptor as detailed in Materials and Methods. In the first round of amplification (20 cycles), an exon 5 specific *Vipr2* primer was used along with the ‘outer’ adapter primer specific for the anchor sequence that was included during the cDNA synthesis, followed by a second round of amplification (20 cycles) using an exon 3 specific Vipr2 primer and the ‘inner’ adapter primer. The position of the 300-bp marker is indicated with an arrow.

The 5′ untranslated region and transcriptional start site for the *Vipr2* gene were determined by rapid amplification of 5′-cDNA ends (5′-RACE) utilising anchored cDNA libraries made from mouse pituitary AtT20 cells and from olfactory bulb tissues isolated from BALB/c and from C57BL/6J mice. This also enabled us to determine if there were additional upstream exons within the gene. Several cDNA clones from the 5′-RACE amplification of the AtT20 cDNA library and from the mouse olfactory bulb cDNA libraries were sequenced. The 5′ ends of all matched the genomic DNA sequence extending upstream of the translational start site in the first coding exon, the longest AtT20 clone and C57BL/6J olfactory bulb clone similarly extending 82 bp upstream. This is flanked at the 5′ end by a putative Initiator (Inr) element (38) in the genomic DNA sequence (Fig. 2a). A second Inr-like element is located downstream from this within exon 1 and flanking the 5′ end of the longest clone amplified from the Balb/c olfactory bulb library (Fig. 2a).

The possibilities of tissue-specific and/or developmentally regulated alternate 5′-transcriptional start sites or alternative splicing of the 5′ region were also examined by 5′-RACE. For this, a RACE-ready panel of anchored cDNA libraries derived from 24 mouse tissues and developmental stages were amplified with the 5′-adapter primer along with a *Vipr2* exon 3-specific oligonucleotide primer, as detailed in the Materials and Methods. The distance of the exon 3-specific primer relative to the 5′ end of the published mouse cDNA sequence (37) is 289 bp. A single major DNA band of approximately 300 bp was detected following gel electrophoresis in eight of the tissues represented in the panel (brain, spleen, stomach, lung, skin, salivary gland and embryonic stages 8–9 days, 9.5 days) (Fig. 2b). These were subcloned and several different cDNA clones sequenced in order to compare with the previously isolated cDNA and genomic DNA sequences. All extended 5′ of the exon 3 specific primer, matching to varying lengths the original published mouse cDNA sequence (37) and the AtT20 and olfactory bulb 5′-RACE cDNA sequences characterised above (Fig. 2a). The longest clone (SRBrain) was amplified from the brain and extended 111 bp upstream of the translational start site, matching genomic DNA sequence that was flanked by a third putative Inr element upstream of the first one identified above (Fig. 2a). This strongly suggests that there is no single transcriptional start site but that transcription can start at various points within an 80 bp region at the 5′ end of exon 1. Several of the various weaker DNA bands were also subcloned and sequenced, but were found to be either nonspecific amplification products having the 5′-adapter primer at both ends or else matched to varying lengths the sequences determined for the other 5′-RACE products (data not shown). None of these extended further upstream than the SRBrain sequence or contained additional sequences that would suggest that upstream exons exist or that the 5′ end is alternatively spliced. Thus, it seems likely that the 80 bp region at the 5′ end of exon 1 constitutes the primary region responsible for transcription of the *Vipr2* gene in mouse.

### Functional analysis of the promoter activities in mouse and rat pituitary cell lines

The sequence of the 5′ region of *Vipr2* was characterised originally from a 5.6-kb *Xba* I genomic DNA fragment isolated from the λES-129 DNA library (Mackay, West, Lutz and Harmar, unpublished data). This fragment includes the ATG initiation codon (exon 1), 3.2 kb of 5′ flanking sequence, intron 1, exon 2 and part of intron 2. To evaluate the promoter activity of the region flanking exon 1, a series of luciferase reporter gene constructs were made by subcloning varying lengths of the 5′ 3.2-kb region in front of the luciferase gene in pGL3 Basic. The adjacent 2.2-kb region spanning the translational start site and part of intron 1 (−32 to +2167) subcloned into pGL3 Basic was also used. These were transiently expressed in two pituitary clonal cell lines that endogenously express the VPAC_2_ receptor, mouse AtT20 D16:16 corticotroph cells and rat GH4C1 somatomammotroph cells, and in COS 7 cells that do not express this receptor (25). Cells were cotransfected with the pRL-TK plasmid containing the *Renilla* luciferase gene under the control of the HSV-thymidine kinase promoter to normalise results between transfections.

**Fig. 3:**
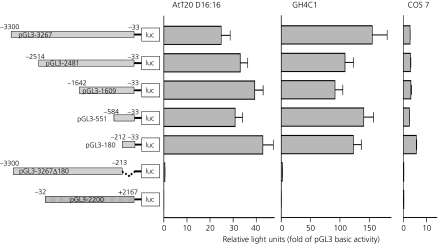
Transient expression of the 5′ flanking region of *Vipr2* in AtT20 D16:16, GH4C1 and COS 7 cell lines. The *Vipr2* sequences present in each luciferase reporter gene construct relative to the ATG start site are indicated on the left. Luciferase activities (firefly and *Renilla*) were measured in cell lysates and normalised to *Renilla* luciferase activity. Results are shown as fold over pGL3 Basic activity (2.6 ± 0.7 rlu; 0.31 ± 0.22 rlu and 1.1 ± 1.1 rlu for AtT20, GH4C1 and COS 7 cells, respectively) and are the means ± SEM of six to nine separate measurements.

The pGL3-3267 construct containing the region spanning 3300 to −33 relative to the ATG start codon gave high levels of luciferase activity when expressed in AtT20 and GH4C1 cells (Fig. 3), 24.9 ± 3.9- and 155.2 ± 25.4-fold of pGL3 Basic activity, respectively. Low levels of luciferase activity were measured when pGL3-3267 was expressed in COS 7 cells (2.9 ± 0.1-fold of pGL3 Basic activity). In comparison, very little luciferase activity was measured in cells expressing pGL3-2200, which contains the adjacent region spanning −32 to +2167 (0.06 ± 0.01-, 0.42 ± 0.2- and 0.2 ± 0.02-fold of pGL3 Basic activity, respectively). The pGL3 Control plasmid containing the SV40 promoter gave 9.1 ± 0.7-, 74.3 ± 34.4- and 66.4 ± 10.2-fold of pGL3 Basic luciferase activity when expressed in AtT20, GH4C1 and COS 7 cells, respectively. Successive 5′ deletions of the 3.2-kb 5′ flanking sequence to a 180-bp region (−212 to −33) maintained high levels of luciferase activity when expressed in AtT20 and GH4C1 cells (Fig. 3). Deletion of this 180-bp region for the pGL3-3267Δ180 (−3300/−213) abolished luciferase activity when expressed in all the cell lines used. Taken together, these results suggest that this 180-bp region forms the core promoter of the *Vipr2* gene.

### Identification of potential transcription factor binding sites

**Fig. 4:**
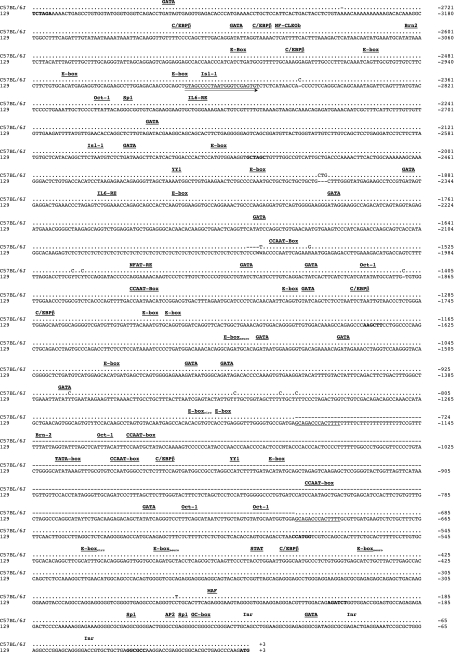
Comparison of the C57BL/6J (accession number AC073564) and 129 (accession number AJ272314) 5′ regions flanking the ATG translational start site of the VPAC_2_ receptor gene. The dots indicate matching sequences, dashes indicate missing sequences. Substitutions and insertions are shown. Putative transcription factor binding sites are labelled above the sequence. The position of the oligonucleotide primer used to amplify the C57BL/6J and BALB/c 5′ flanking regions is underlined. The *Sfo*I, *Nhe*I, *Hind*III, *Nco*I and *Bgl*II restriction sites used to make the 5′ flanking region luciferase reporter gene constructs are shown in bold.

Analysis of the 3.2-kb 5′ flanking region using the transcription factor databases TRANSFAC 3.0 including MatInspector 2.0 (GBF, Braunschweig, Germany) (39), and TFD (National Institute of Health, Bethesda, MD, USA) identified several potential transcription factor binding sites that may be involved in the tissue- and developmental-specific and inducible expression of *Vipr2*. The 180-bp core promoter region is extremely GC-rich (the GC content is 70%), and contains in addition to the three Inr-like elements binding sites for Sp1 and AP2. Within 500 bp of exon 1 are three E-box elements, including a noncanonical E-box motif for circadian regulation (40) and two E-box motifs for the NeuroD1/Beta2 factor (41) (Fig. 4). In addition, there are STAT, NF-IL-6 and C/EBPβ binding sites. Further upstream are several E-box and GATA elements, a NF-CLEOb site, as well as sites for YY1, Oct – 1, Brn-2, Isl-1 and C/EBPβ (Fig. 4).

### *L1(LINE-1) polymorphism in the mouse Vipr2 promoter*

**Fig. 5:**
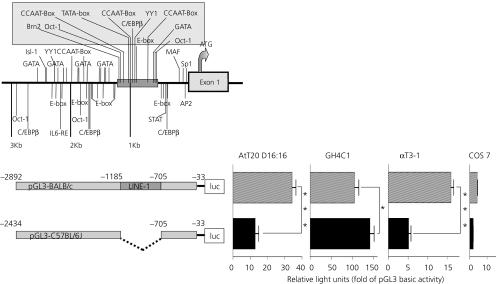
Transient expression of the *Vipr2* 5′ flanking sequences derived from BALB/c and C57BL/6J VPAC_2_ receptor genes in AtT20, GH4C1, αT3-1 and COS 7 cells. The positions of the L1 sequence and potential transcription factor binding sites are indicated in the schematic diagram of this region in the upper left. Luciferase activities (firefly and *Renilla*) were measured in cell lysates and normalised to *Renilla* luciferase activity. Results are shown as fold over pGL3 Basic activity (2.6 ± 0.7 rlu; 0.31 ± 0.22 rlu, 0.38 ± 0.05 rlu and 1.1 ± 1.1 rlu for AtT20, GH4C1, αT3-1 and COS 7 cells, respectively) and are the means ± SEM of six to nine separate measurements (***P < 0.001, *P < 0.05, one-tailed Student t-test).

Lying 705 bp upstream of the translational start site of the mouse *Vipr2* gene, we have discovered a 464-bp DNA sequence that bears a significant similarity to the mouse L1 family, flanked on each end by a 16-bp terminal repeat sequence. So far, this L1-like element has been found in the *Vipr2* promoter derived from 129 and Balb/c genomic DNA but is not present in the *Vipr2* promoter from C57BL/6J (Fig. 4). It contains various putative *cis*-acting regulatory elements, including a TATA-box, three CCAAT-boxes and binding sites for the transcription factors Oct-1 and Brn-2, as well as E-box, GATA, YY1 and C/EBPβ sites (Fig. 4), which suggests that the L1-like sequence may have a direct effect on *Vipr2* promoter activity. Conversely, this polymorphism may have a disruptive effect on the organisation of the promoter's transcriptional machinery. To test whether the polymorphism is functionally important, cells were transfected with luciferase reporter gene constructs containing the *Vipr2* promoter region with or without this sequence. These were made with the 2.9 kb Balb/c (−2892/−33) or the 2.4-kb C57BL/6J (−2434/−33) *Vipr2* gene 5′ flanking sequences, respectively. A significant increase in luciferase activity was observed for both reporter constructs when expressed in AtT20 and GH4C1 cells (Fig. 5), indicating that they are functional promoters. The pGL3-BALB/c construct gave similar levels of luciferase activity as the 129 genomic DNA construct pGL3-2481 (Fig. 3) when expressed in each of the cell lines. However, the pGL3-C57BL/6J showed three-fold less activity than pGL3-BALB/c when expressed in AtT20 cells, but not when expressed in GH4C1 cells (Fig. 5). To determine whether this was cell-specific, both reporter gene constructs were expressed in the mouse pituitary αT3-1 gonadotroph cell line, which also expresses the VPAC_2_ receptor but at low levels (21). The pGL3-C57BL/6J showed three-fold less activity than pGL3-BALB/c when expressed in αT3-1 cells as well, indicating that there may be species- rather than cell line-differences between the two promoter constructs.

## Discussion

In the present study, we have characterised the structure of the mouse VPAC_2_ receptor gene, *Vipr2*, and have identified the transcriptional start sites and the core promoter region. The relationship between the intron/exon structure of the mouse *Vipr2* and the transmembrane topology that it encodes is highly homologous to the structure of the human gene (35), and is similar to that reported for other members of the secretin receptor gene family (42–49). All of the exon/intron boundaries conform to the consensus GT-AG rule for splice junctions (50). Primer extension was tried in the first instance to determine the transcriptional start site, but was unsuccessful, possibly due to the high GC content and also to there being multiple sites where transcription can be initiated. Analysis of the 5′ UTR of several cDNA clones amplified from an anchored AtT20 cDNA library and from anchored olfactory bulb cDNA libraries from Balb/c and C57BL/6J mice indicated that transcription can be initiated at multiple sites. The first was identified from the longest clone amplified from the AtT20 library (and corresponding to that amplified from the C57BL/6J olfactory bulb library) that extended the 5′ end of exon 1 to 82 nucleotides upstream of the ATG translational start site. A second site was identified within exon 1 from the longest clone amplified from the Balb/c olfactory bulb cDNA library that extended 43 nucleotides upstream of the ATG translational start site. Each is flanked on the 5′ end by an 8-bp sequence that resembles an Inr element (pyrimidine, pyrimidine, A(+1), N, T/A, pyrimidine, pyrimidine) (38), with that at the first site (−89 to −82) differing by only one nucleotide. At the end of each of these Inr-like motifs is the TG dinucleotide, previously defined as one of the three most preferred initiators by a genome-wide screen (51).

For some members of the secretin receptor gene family, tissue- and developmentally-specific alternative promoters and/or splicing of the 5′ untranslated regions have been reported (52–57). In addition, the sequence of a human prostate cDNA clone encoding a VPAC_1_ receptor with an altered N-terminal sequence that would arise from a 5′-alternatively spliced transcript has been reported in the database (58), although whether this transcript is functionally expressed has not yet been determined. The possibility that such events play a role in the regulation of the *Vipr2* gene was examined here using 5′-RACE PCR analysis of a panel of anchored cDNA libraries derived from different mouse tissues and developmental stages. Several cDNAs were sequenced, the longest extending 111 bp upstream of the ATG translational start site and extending the 5′ boundary of exon 1. This also was flanked at the 5′ end by an Inr-like element located at −120 to −113 in the genomic DNA sequence, thus identifying a third possible transcriptional start site. There was no evidence that further exons existed upstream of exon 1 or that the 5′ end is alternatively spliced, as the 5′ end of all of the 5′-RACE cDNA clones that were sequenced matched that of exon 1. For two genes that are alternatively spliced in the 5′-UTR, the rat PAC_1_ receptor gene and the mouse PTHR1 gene, a consensus 3′ splice acceptor site is present a short distance (76 bp and 45 bp, respectively) upstream of the translation start codon (52, 53). No potential splice acceptor sites corresponding to consensus splice site sequence (3′ Y_11_NYAG,G) (59) are found within the corresponding region of the mouse *Vipr2* gene. Furthermore, we have determined previously that the 187 bp 5′-UTR (sequenced from the longest 5′-RACE product amplified from a human placental cDNA library) and the translational start site of the human gene are encoded within the same exon (35). Thus, it is likely that exon 1 contains both the major sites for initiation of transcription as well as for translation for the mouse *Vipr2* gene.

The core promoter region for the mouse *Vipr2* gene was identified as a 180 bp sequence located at −212 to −33 relative to the ATG initiation codon. A CpG island begins 160 bp upstream of the translational start site and extends 375 bp into intron 1 of *Vipr2*. As for the human gene (35), no TATA- or CCAAT-box sequences are found proximal to exon 1 for the mouse *Vipr2* gene. Instead, consensus binding sites for the transcription factor Sp1 and related factors are present. These, in addition with a broad range of transcriptional start sites, are key features of CpG island promoters (51, 60). Although CpG island promoters are mainly associated with house-keeping genes, they are also present in the promoter regions of a significant proportion of genes that show tissue-specific expression (51, 61). Interestingly, there is increasing evidence that genes that are active during early development contain CpG island promoters (62). CpG island promoters may be a common feature for genes encoding the Group II secretin receptor GPCRs. The promoter regions that have been characterised for the secretin receptor gene family are typically GC-rich, and many have Sp1 sites but not a TATA-box upstream of the transcriptional start site (45, 48, 52, 57, 63–69). Sp1 sites provide an alternative mechanism to initiate the transcription of genes that do not contain a TATA-box in their promoter region (70). The Sp1 sites have been shown to be active for the rat VPAC_1_ receptor (65), PTH receptor (67), mouse glucagon receptor (68) and human secretin receptor genes (69).

The mouse and rat *Vipr2* promoter regions are highly conserved, comparison of the 4.5-kb 5′ region flanking exon 1 between the mouse (C57BL/6J) and the rat (accession number AC096277) genes shows 78% identity. There is much less conservation between the mouse and human (accession number Y18423) 5′-flanking and exon 1 sequences, the 650-bp regions proximal to exon 1 have 45% identity between the mouse and human genes. However, the overall structural features of both promoter regions are similar. Both have clusters of GATA and E-box sequences as well as C/EBPβ binding sites within 3 kb of the translational start site that may be functionally significant for the tissue-specific and developmental expression of the gene in human and mouse. Several E-box sequences that are contained within both promoter regions are consensus sites for the basic helix-loop-helix factor NeuroD1/Beta2 (41). Within the human *VIPR2* promoter, there are 25 NeuroD1/Beta2 sites, 24 of which are within the GATA repeat region (35). C/EBPβ and NeuroD1/Beta2 are important for neurogenesis (71, 72) and the development of different pituitary cell lineages is dependent on both NeuroD1/Beta2 and GATA-2 factors (73). Thus, the presence of these sites within the human and mouse promoter regions suggests that the VPAC_2_ receptor may potentially have a role in these processes in both species. Two E-box elements within the mouse *Vipr2* promoter are binding sites for the circadian regulators BMAL/CLOCK (40, 74) and may be responsible for the oscillatory modulation of transcription for the VPAC_2_ receptor gene in mice (75). The circadian regulator E-box elements were not found in the corresponding human *VIPR2* promoter region.

Lying within the 5′ flanking sequence of the mouse *Vipr2* gene, we have discovered a 496-bp polymorphic DNA sequence that bears a significant homology to mouse L1 DNA. This sequence is present in the *Vipr2* promoter regions in 129 and BALB/c but not in C57BL/6J. L1 elements constitute the most common family of long interspersed elements in the mammalian genome (76). These mobile genetic elements form part of the retrotransposon family and are characterised by the presence of flanking direct repeat sequences. The self-propagating nature of L1s is due to the presence of two ORFs, which encode an RNA-binding protein, an endonuclease, reverse transcriptase and a highly conserved cysteine-rich motif, all of which are required for reverse transcription of the L1 element via an RNA intermediate. Although full length LINEs are normally approximately 6–7 kb, L1 elements are often found as inactive truncated versions which are incapable of retrotransposition (77). The function of L1 elements remains elusive but it appears that these molecular parasites exist only to maintain themselves and their presence in the genome is far from insignificant. We have shown here that the L1 sequence within the promoter region of the *Vipr2* gene confers a three-fold increased level of expression for the luciferase reporter gene construct pGL3-BALB/c compared to pGL3-C57BL/6J containing the equivalent region without this sequence when transiently expressed in two mouse pituitary cell lines. The simplest explanation for this observation is that elements within the L1 sequence may enhance expression of the gene. However, this appears unlikely as the luciferase reporter gene construct pGL3-551, in which the 5′ region, including the L1 sequence, was deleted, maintained similar levels of expression compared to the pGL3-BALB/c construct. A more likely explanation is that the region upstream of the L1 sequence contains a repressor element (or elements) and that the L1 sequence contains an element that binds a transcriptional regulator that ‘relieves’ this repression. One such candidate is the multifunctional transcription factor Yin Yang-1 (YY1), which can act as a transcriptional repressor, activator or initiator, depending on the context of its binding site in relation to those of other transcription factors (78). For the *Vipr2* gene, a YY1 repressor site is located approximately 1.25 kb upstream of the L1 sequence and a YY1 activator site is located midway within the L1 sequence. However, there are several elements within the L1 sequence that may contribute to the enhanced level of expression that is observed and we are currently investigating these to ascertain which are relevant.

In summary, the exon/intron organisation of the mouse VPAC_2_ receptor gene, *Vipr2*, is highly conserved with that of the human gene and with other genes encoding Group II secretin receptor GPCRs. Transcription of *Vipr2* is initiated from multiple sites within an 80 bp region at the 5′ end of exon 1 and the core promoter region is contained within the GC-rich region proximal to the ATG translational start site. Several regulatory sites located upstream of the core promoter region were identified that may contribute to the tissue-specific, inducible and developmentally regulated expression pattern observed for *Vipr2* mRNA. In addition, a polymorphic L1-like sequence was identified that is present in the *Vipr2* promoter regions of 129 and BALB/c mice, but not in C57BL/6J, that could potentially contribute to differential expression levels of the gene. The functional consequences of this remain to be explored.
